# The Cochrane Collaboration: A leading role in producing reliable evidence to inform healthcare decisions in musculoskeletal trauma and disorders

**DOI:** 10.4103/0019-5413.41849

**Published:** 2008

**Authors:** Helen H Handoll, William J Gillespie, Lesley D Gillespie, Rajan Madhok

**Affiliations:** School of Health and Social Care, University of Teesside, Middlesbrough, UK; 1 Hull York Medical School, University of Hull, Cottingham Road, Hull, UK; 2 Department of Medical and Surgical Sciences, Dunedin School of Medicine, University of Otago, Dunedin, New Zealand; 3 School of Translational Medicine, University of Manchester, Manchester, UK

**Keywords:** Evidence-based medicine, meta-analysis, orthopedics, randomized controlled trials

## Abstract

Systematic reviews are a key component of evidence-based practice. A valuable and accessible source of good quality systematic reviews on topics in musculoskeletal trauma and disorders is the Cochrane Database of Systematic Reviews, published in *The Cochrane Library*. These reviews are produced by members of The Cochrane Collaboration, an international not-for-profit organization that aims to make up-to-date, accurate information about the effects of healthcare readily available worldwide. Contributions from orthopedic specialists in India and neighboring countries are required to make the Cochrane Database an even more useful and comprehensive resource of reliable evidence. Linked with this is the opportunity for orthopedic specialists to take a leading role in generating the evidence to inform their practice.

## INTRODUCTION

The large amount of information available in paper journals and electronically means that it is impossible for any single clinician to access, let alone assimilate, the primary evidence to reliably inform everyday practice decisions. However, access to systematic reviews and other evidence-based summaries based on systematic reviews (e.g. evidence-based guidelines) can make this easier.

Systematic reviews are a keystone of evidence-based medicine. Systematic reviews of randomized controlled trials constitute the top level of evidence for the effectiveness of healthcare interventions because they are more likely to provide valid (less biased) evidence of the effectiveness of the trial interventions.^1^–^5^ Starting from a clearly defined research question, such reviews use systematic, predefined and explicit methods to identify, select and critically appraise all relevant research, collect and analyze data from eligible studies, and to present results and draw conclusions. Where statistical techniques are used to combine, or pool, the results of the included studies, systematic reviews are often called meta-analyses. Systematic reviews can also inform the research agenda by identifying gaps in the evidence, generating research questions and informing study design, conduct and reporting; they are a prerequisite for grant applications for primary research and related reports for publicly funded bodies in several counties including UK, the Netherlands and Canada.

Over the last decade, the majority of systematic reviews have addressed questions about the effectiveness of treatment regimens or strategies. Randomized trials comparing diagnostic strategies have been uncommon. However, methods for the systematic review and meta-analysis of cohort and case-control studies evaluating the effectiveness of diagnostic tests are emerging, and diagnostic reviews are likely to become more important in the next few years.

This article describes an important and accessible source of high-quality systematic reviews, the Cochrane Database of Systematic Reviews, which is the major product of an international organization called The Cochrane Collaboration. It also describes the role of the Cochrane Review Groups that contribute reviews relating to musculoskeletal injuries and conditions. It concludes by observing the potential for the orthopedic community in India to make an important contribution to this international endeavor and also to play a leading role in generating the evidence.

## THE COCHRANE COLLABORATION

### The Cochrane Library

The Cochrane Database of Systematic Reviews is one of seven databases available in *The Cochrane Library*, an electronic publication which can be considered the key resource for information on the effectiveness of healthcare interventions. *The Cochrane Library* is currently published electronically on a quarterly basis. Another notable and unique database in *The Cochrane Library* is the Cochrane Central Register of Controlled Trials (‘Clinical Trials’; CENTRAL) which has over half a million references to controlled trials. National subscriptions of several counties endorse the value of *The Cochrane Library*. In India national access to the full-text version for all residents was gained in February 2007, funded by the Indian Council of Medical Research [Box 1].

Box 1 **Access to *The Cochrane Library* (and Cochrane Database of Systematic Reviews)**

- Free access to abstracts and summaries of Cochrane Reviews are available from: www.thecochranelibrary.com and www.cochrane.org
- India: access is available via: www.icmr.nic.in (click on The Cochrane Library link - currently (May 2008) bottom left hand side of home page of The Indian Council of Medical Research)

Later in 2008, Cochrane Reviews of Diagnostic Test Accuracy will be introduced into *The Cochrane Library*. Currently, protocols for eight pilot reviews, two on musculoskeletal conditions, are available for viewing via the website. This promises to be a very important development.

### The Cochrane Database of Systematic Reviews

This database is the most comprehensive full-text source of up-to-date systematic reviews of healthcare interventions available worldwide. The size and coverage of this database grows with each new issue: the second issue in 2008 contained 3464 Cochrane reviews, of which 80 were entirely new and 52 were updated reviews (all involving a new search and, in 36 of these, changed conclusions). In addition, there were 1856 protocols for future reviews. The majority of Cochrane reviews include only randomized or quasi-randomized trials. It is this restriction and other steps, such as drawing from the massive international effort to identify relevant studies, and use of explicit, predefined methods used to minimize bias in the review process, that make Cochrane reviews an especially reliable source of evidence. The regular updating of Cochrane reviews should ensure their continued relevance.

### The Cochrane Collaboration

The Cochrane Database of Systematic Reviews is the main product of The Cochrane Collaboration, which is an international not-for-profit and independent organization, dedicated to making up-to-date, accurate information about the effects of healthcare readily available worldwide. It produces and disseminates systematic reviews of healthcare interventions and promotes the search for evidence in the form of clinical trials and other studies of interventions. The Collaboration was founded in 1993 and named after the British epidemiologist, Archie Cochrane who, in 1979, identified the need for organizing “a critical summary, by specialty or subspecialty, adapted periodically, of all relevant randomized trials”.^4^

The work of the Collaboration is based on this shared vision held by over 13,000 active contributors, mainly working on a voluntary and unpaid basis, worldwide. The 10 principles that guide this vision are: collaboration; building on the enthusiasm of individuals; avoiding duplication; minimizing bias; keeping up-to-date; striving for relevance; promoting access; ensuring quality; continuity; and enabling wide participation. For further information see the Collaboration's website (www.cochrane.org).

### Cochrane Review Groups

The main work of the Collaboration is carried out by members of 51 Cochrane Review Groups (CRGs), each of which concentrates on a specific aspect of healthcare. Members of these Groups includes healthcare professionals, researchers, people using healthcare services (consumers), and others who have come together because of their shared interest and commitment to produce reliable up-to-date summaries of the evidence relevant to the prevention, treatment and rehabilitation of particular health problems. Each Group has an editorial team overseeing the preparation and maintenance of reviews, as well as the application of rigorous quality standards. This team is supported by a staff member (Review Group Coordinator or Managing Editor) who is paid to organize and manage the day-to-day activities of the Group. Usually, an Information Retrieval Expert (Trials Search Coordinator) is also employed to assist with the identification of trials relating to the Group's scope. These trials are also listed in *The Cochrane Library* in the ‘Clinical Trials’ database.

Brief summaries of the three CRGs focusing on musculoskeletal conditions, and their contact details are provided in Table 1. Inevitably, there are areas of overlap in the scopes of these three CRGs and with other CRGs. Given the essential ethos of collaboration between Groups, this in fact poses no practical problems for users or contributors to the Cochrane Database of Systematic Reviews. Details of other CRGs with potentially relevant topics, such as the Cochrane Anesthesia Group, Injuries Group, and Wounds Group, are available on the Cochrane website (www.cochrane.org/contact/entities.htm#CRGLIST).

**Table 1:** Scopes and contact details of the three Cochrane Review Groups focusing on musculoskeletal conditions

Back Group	Bone, Joint and Muscle Trauma Group	Musculoskeletal Group
**Scope**	**Scope**	**Scope**
Areas of primary and secondary prevention and treatment of neck and back pain and other spinal disorders, excluding inflammatory diseases and fractures.	1. The primary and secondary prevention of fractures, dislocations, and soft tissue injuries of the appendicular skeleton and associated soft tissues, and the prevention of complications or adverse effects of treatment;	Musculoskeletal disorders in the following categories: Gout, Legg Calve Perthes, lupus erythematosus, ostearthritis, osteoporosis, pediatric rheumatology, rheumatoid arthritis, soft tissue conditions, fibromyalgia, spondylo-arthropathy, systematic sclerosis, vasculitis.
	2. The management (treatment and rehabilitation) of people with fractures, dislocations, and soft tissue injuries of the appendicular skeleton and associated soft tissues.	
**Contact:** Victoria Pennick, Institute for Work and Health, 481 University Avenue, Suite 800, Toronto Ontario M5G 2E9, Canada, Phone:+1 416 927 2027 ext: 2158, Fax:+1 416 927 4167, Email: vpennick@iwh.on.ca, Group's website: www.cochrane.iwh.on.ca	**Contact:** Lindsey J Elstub, Epidemiology Research Group, School of Translational Medicine, The University of Manchester, 2^nd^ Floor Stopford Building, Oxford Road, Manchester, M13 9PT, UK, Phone:+44 161 2755953, Fax:+44 161 2755043, Email: lindsey.elstub@manchester.ac.uk, Group's website; www.bjmtg.cochrane.org	**Contact:** Lara Maxwell, Institute of Population Health, University of Ottawa, 1 Stewart Street, Ottawa Ontario K1N 6N5, Canada, Phone:+1 613 562 5800 ext: 1977, Fax:+1 613 562 5659, Email: cmsg@uottawa.ca, Group's website: www.cochranemsk.org

### Other Cochrane entities

Other Cochrane entities include the 12 Cochrane Centers around the world. One of their core functions is the coordination of training and support for review authors in their ‘catchment’ area. Several centers have ‘branches’; these include the South Asian Cochrane Network of the Australasian Cochrane Centre, which was established in December 2004. The network includes the following countries: Bangladesh, Bhutan, India, Maldives, Nepal, Pakistan and Sri Lanka. The Network consists of a central coordinating base in Vellore, India and is a devolved network of sites across the South Asian region, each contributing to and supporting the activities of SACN. Website: www.cochrane-sacn.org

### Ways of contributing to the Cochrane Collaboration

The participation of orthopedic surgeons in the work of the Collaboration is important, not only in the creation of new reviews, but, increasingly important as the Cochrane Database of Systematic Reviews grows in size, in keeping the reviews up-to-date and relevant. Three main ways of direct involvement are as a review author, an editor (note, the successful completion of a Cochrane review is mandatory for this role) and a hand searcher of journals. This later role involves searching medical journals for accounts of controlled trials that are not yet indexed in major electronic databases such as MEDLINE. Though time-consuming, participating as a review author is particularly rewarding as it offers an opportunity to share your expertise with an international audience as well as being part of a large international organization.

Direct sustained contributions in these ways may not be possible for many busy healthcare professionals, but other important contributions can be made with less of a time commitment. Specifically, all protocols and reviews are peer refereed, and more content experts are required to fulfill this role to ensure the quality and relevance of the reviews.

## THE COCHRANE BONE, JOINT AND MUSCLE TRAUMA GROUP (BJMTG)

This Group, formerly the Musculoskeletal Injuries Group, was set up in 1994 with its editorial base in Edinburgh, UK. Currently, the editorial base is in Manchester, UK. The Group has around 300 members from numerous countries and has been pleased to receive a number of recent expressions of interest from orthopedic surgeons in India.

As of May 2008, the Group has 80 published reviews, 25 published protocols for future reviews and 17 registered titles being developed into protocols (see: www.bjmtg.cochrane.org; ‘Our reviews’). The size and scope of these reviews varies enormously. As evidence accumulates, some large reviews are being split into several more manageable but still clinically appropriate reviews.

Systematic reviews are available for several of the most common musculoskeletal injuries. For example, the Group's members have produced reviews covering most aspects of the management of three key osteoporotic fractures: hip, distal radius and proximal humerus. As shown in Table 2, completed reviews for hip fracture span the care pathway from initial treatment through to the prevention of fracture recurrence. However, given the extensive scope of the Group, systematic reviews for many other injuries are not available: there is much more work to be done.

**Table 2:** Cochrane reviews on the management of patients with hip fractures produced by the Cochrane Bone, Joint and Muscle Trauma Group

Area	Review topics*
Initial treatment	Nerve blocks Preoperative traction
Prevention of complications	Antibiotic prophylaxis for surgery Heparins and physical methods for preventing thrombosis following surgery Non-steroidal anti-inflammatory drugs for preventing heterotopic bone formation after arthroplasty
Choice of treatment	Conservative versus operative
Choice of implant	Arthoplasties (with and without bone cement) Condylocephalic nails versus extramedullary implants (extracapsular fractures) Extramedullary fixation implants and external fixators (extracapsular fractures) Cephalocondylic intramedullary nails versus extramedullary implants (extracapsular fractures) Internal fixation implants (intracapsular fractures) Internal fixation versus arthroplasty (intracapsular fractures) Intramedullary nails (extracapsular fractures) Replacement arthroplasty versus internal fixation (extracapsular fractures)
Surgical technique	Surgical techniques for internal fixation (extracapsular fractures) Surgical techniques for internal fixation (intracapsular fractures) Surgical techniques for hemiarthroplasty
Choice of anesthesia	Methods of anesthesia
Postoperative care and rehabilitation	Wound drainage Coordinated multidisciplinary inpatient rehabilitation Mobilization strategies Nutritional supplementation
Prevention of re-fractures	Fall prevention Hip protectors Vitamin D and vitamin D analogues

* Reviews are for all types of hip fracture unless indicated

The time and resources invested in generating these and other Cochrane reviews is only worthwhile if they are valued and their findings used by patients, health professionals, and those charged with funding and managing health systems. There is increasing evidence that this is the case. For example, BJMTG reviews have made major contributions to a number of evidence-based guidelines and summaries, such as those for hip fracture.^6^–^8^ Many reviews have been used to justify primary research, including large multicenter trials.^9^,^10^

## INDIA AND THE DEVELOPING WORLD PERSPECTIVE

In many areas of orthopedic practice, including the management of injuries, the number of randomized trials which have been conducted is disappointingly small. There have also been concerns about the quality of primary research.^11^,^12^ In the past these have limited the impact of systematic reviews in the developed world, although that is now changing.^1^ For the developing world, these limitations are intensified. Two fundamental considerations are the major differences in the epidemiology of injury and injury-related conditions, and in the provision of healthcare. For example, although changing demographics mean that the predicted growth in hip fracture is in Asia,^13^ fractures resulting from road traffic accidents and other high-energy trauma remain dominant in many developing countries with predominantly young populations. Poverty and inadequate access to healthcare not only affect the presentation of injury but also, patently, strongly influence treatment choice and availability. Overall, primary orthopedic research of direct relevance to the developing world is relatively sparse. Clearly, even where treatment options are relatively few and basic there is a mandate for evidence-based orthopedics and, by implication, a need for primary research where reliable evidence is lacking.

In particular, India's rapid economic growth presents a remarkable opportunity for its orthopedic community to develop a consensus on those research questions which are important at the current stage of development of the health system and health technology, but for which little or no high-quality evidence exists. Our experience in moving towards a research agenda for distal radial fractures may offer some insights on the prioritization of research questions.^14^ Injuries are “substantially underrepresented in the relative proportion of quality-adjusted original research output as compared with their contribution to the disease burden”.^15^ The proposal to develop a collaborative trials network in India^16^ could bring Indian orthopedics to the forefront of orthopedic research, if it can lead to the successful promotion and conduct of high-quality clinical trials at both the technical end of the spectrum of orthopedic practice, and in those communities where the improvement of healthcare will initially take place at a less sophisticated level. Allied to this is the imperative for the ongoing systematic review of current and accumulating evidence.

## CONCLUSION

In this article, we have described the work of The Cochrane Collaboration, many of whose reviews, freely available on the web in numerous countries including India, will be relevant to the reader's practice. But there are many gaps in the evidence, some reflecting missing topics, and others missing perspectives. Thus, as well as using evidence to inform practice, the readers of this article should consider how they can address the deficiencies in the evidence needed to inform orthopedic practice. To do this they should take heed of the national calls for change^16^,^17^ and the establishment of the Clinical Trials Registry - India (www.ctri.in) and the South Asian Cochrane Network. The orthopedic community in India has the opportunity and the potential to make a difference and become a major force in evidence-based orthopedics.
